# Exploring the influence of context and policy on health district productivity in Cambodia

**DOI:** 10.1186/s12962-016-0051-6

**Published:** 2016-01-22

**Authors:** Tim Ensor, Sovannarith So, Sophie Witter

**Affiliations:** International Health Systems, Leeds Institute of Health Sciences, Leeds, UK; Cambodia Development and Research Institute, Phnom Penh, Cambodia; International Health Financing and Systems, IIHD, Queen Margaret University, Edinburgh, UK

**Keywords:** Data envelopment analysis, Regression analysis, Health districts, Productivity, Efficiency, Cambodia, Health sector reform

## Abstract

**Background:**

Cambodia has been reconstructing its economy and health sector since the end of conflict in the 1990s. There have been gains in life expectancy and increased health expenditure, but Cambodia still lags behind neighbours One factor which may contribute is the efficiency of public health services. This article aims to understand variations in efficiency and the extent to which changes in efficiency are associated with key health policies that have been introduced to strengthen access to health services over the past decade.

**Methods:**

The analysis makes use of data envelopment analysis (DEA) to measure relative efficiency and changes in productivity and regression analysis to assess the association with the implementation of health policies. Data on 28 operational districts were obtained for 2008–11, focussing on the five provinces selected to represent a range of conditions in Cambodia. DEA was used to calculate efficiency scores assuming constant and variable returns to scale and Malmquist indices to measure productivity changes over time. This analysis was combined with qualitative findings from 17 key informant interviews and 19 in-depth interviews with managers and staff in the same provinces.

**Results:**

The DEA results suggest great variation in the efficiency scores and trends of scores of public health services in the five provinces. Starting points were significantly different, but three of the five provinces have improved efficiency considerably over the period. Higher efficiency is associated with more densely populated areas. Areas with health equity funds in Special Operating Agency (SOA) and non-SOA areas are associated with higher efficiency. The same effect is not found in areas only operating voucher schemes. We find that the efficiency score increased by 0.12 the year any of the policies was introduced.

**Conclusions:**

This is the first study published on health district productivity in Cambodia. It is one of the few studies in the region to consider the impact of health policy changes on health sector efficiency. The results suggest that the recent health financing reforms have been effective, singly and in combination. This analysis could be extended nationwide and used for targeting of new initiatives. The finding of an association between recent policy interventions and improved productivity of public health services is relevant for other countries planning similar health sector reforms.

**Electronic supplementary material:**

The online version of this article (doi:10.1186/s12962-016-0051-6) contains supplementary material, which is available to authorized users.

## Background

Since the end of the conflict in Cambodia, Gross Domestic Product per capita has increased from $316 US in 1996 to $946 US in 2012 [1]. Health expenditure has also risen from $23 US per capita in 1996 to $69 US in 2012 [2]. The life expectancy of Cambodians has improved, rising from 54 to 61 years for men and 58 to 64 years for women over 2000–2008 [3]. The maternal mortality ratio has fallen from 690 to 290 while the infant mortality rate has declined from 95 to 45 deaths per 1000 live births over the period of 2000–2010 [4, 5]. Despite these improvements, outcomes remain inferior to other Asian countries particularly given that health spending per capita is one of the highest in the region [6].

In an attempt to increase access to health services and improve health outcomes, the government established the operational district (OD) as a focus for health services in 1996. ODs grouped existing administrative districts to form a network of health centres and referral hospitals covering between 100,000 and 200,000 people. Services, staffing requirements and management systems were defined by operational guidelines with the aim of promoting universal coverage across the country [7, 8]. Achievement of universal health coverage is dependent on the effective performance of the OD with adequate resources [9].

To improve their effectiveness, a number of health development initiatives have been introduced over the period with the aim of increasing motivation of health workers and utilisation of facilities, particularly in rural areas. Demand-side schemes to remove access barriers to the poor have been introduced since the mid-1990s, including formalising charges in public facilities (1996), Community-based Health Insurance (CBHI) in 1998; Health Equity Funds (HEF), a subsidy system to increase access for poor people in 2000 [10–12]; and the government National Midwifery Incentive Scheme (NMIS) and vouchers for deliveries, both in 2007 [13]. Income from demand-side schemes are pooled into the facility revenues and largely used to provide salary supplement for staff and administrative operating costs [14]. Supply-side mechanisms have also been introduced to strengthen human resource and facility management, including contracting-in and out of health management and service delivery working with international NGO operators between 1999 and 2002, a hybrid contracting implemented between 2003 and 2008, and internal contracting in form of Special Operating Agencies (SOA) since 2009 [9].

SOAs are ODs which are assessed as having met the capacity criteria and which are therefore granted greater autonomy over financial and other resources. The Ministry of Health and Provincial Health Departments commission services from them, which are funded through Service Delivery Grant (SDG) backed by donor pooled funds. The performance based contracting is based on planned outputs and staff performance indicators. SOAs have been adopted and scaled up from 11 ODs in 2009 to 22 ODs out of a total of 77 in 2012. The SOA roll-out has been supported by pooled funds from seven donors, including the World Bank, DFID, AUSAID, UNFPA, UNICEF, ADB and BTC [15]. With their ability to scale up financial incentives and adopt results-based management, utilisation of public health facilities was expected to increase and become more efficient. However, increasing productivity of public health services and improvement of OD operations through adopting and taking ownership of those health development initiatives remains very much debated. So far little attention has been paid to assessing the change in overall productivity of ODs and its determinants. That is the focus of this article.

The analysis was undertaken as part of a research programme examining health system reconstruction post-conflict, within which one component focussed specifically on the changing incentive environment for health workers, including changing productivity [16]. We use data envelopment analysis (DEA), a non-parametric technique for estimating a production frontier which has been extensively used across public and private sectors to assess relative efficiency of individual firms and industries [17]. The method has been used widely in the health sector in high and low income countries. DEA has been used to investigate the efficiency of inpatient care across a range of countries including Costa Rica, Namibia, Kenya and Zambia [18–21]. Less use has been made of the technique at the primary care level, although there have been studies in Zambia, Sierra Leone and Pakistan [22–24]. Little use has been made of the technique to analyse efficiency of services across a network of facilities and there appear to be no published studies from Cambodia. In most cases, studies focus on outputs from health care including inpatient-days, outpatients and, rarely, patients treated adjusted for diagnosis. Second stage analysis to analyse the impact of contextual variables such as facility ownership, socio-economic conditions and geography is frequently carried out. There appear to be few attempts to use DEA to understand the association between specific health reforms and changes in efficiency. Our article therefore adds both to understanding of productivity in Cambodia and also extends the use of the DEA methodology.

## Methods

This article assesses the determinants of productivity of OD services in five provinces. The analysis makes use of data envelopment analysis (DEA) to measure the efficiency of each operational district in utilising resources to produce health services relative to other areas. The productivity of operational districts, defined as changes in efficiency over time, is analysed using a Malmquist total factor productivity index. Efficiency estimates are analysed by Tobit regression to assess the association with the implementation of health policies.

DEA has a number of advantages over regression-based techniques such as stochastic frontier analysis, including not having to specify a functional form for the production function and permitting the modelling of multiple outputs. The main weakness of the method is that, unlike stochastic regression methods, there is no test of significance and so no guide to the quality of results. It is suggested that it be used principally as an exploratory tool “rather than as an instrument with which to extract precise estimates of organisational efficiency” [25]. As with many quantitative measures of efficiency, the analysis makes no allowance for the quality or outcome of the outputs. It could be the case, for example, that those facilities that deliver lower value in terms of outputs per input(s) may be delivering a higher quality service that leads to better patient outcomes.

The focus of the study is the operational district over a period of 4 years from 2008 to 2011. This period was defined by data availability but includes a period when many of the reform initiatives described earlier were being introduced (see Table 1). Data on 28 operational districts were obtained, focussing on the five provinces that are representative of a range of conditions in Cambodia (geographic conditions, urban/rural populations, and also different levels of external investment). Phnom Penh was included in the wider study but removed from this analysis because of the gaps in information on staffing and limited participation in key informant interviews (Table 2).

**Table 1:** Coverage of health initiatives in target provinces and ODs

	OD	Delivery vouchers^a^	User fees formalised	HEF/CBHI	National maternity incentives scheme	Contracting-in and out		SOA (SDG)
		2012	1997–2011	2012	2007–present	1999–2003	2004–2008	2009–2010
Battambang^a^	5	3	5	5	5			
Kompong Cham	10	9	10	10	10	5	5	5
Kandal^b^	8	0	8	2	8			
Kompot^b^	4	3	4	3	4			
Stung Treng	1	0	1	1	1			

Source: MoH Updated Administrative Records

^a^In Battambang, delivery vouchers started in 2008 in three ODs and building up HEF experiences started in 2006 for some health centres in three ODs to scale up implementation for all five ODs in 2011

^b^Two OD in Kampot (Angkor Chey, Kampong Trach and Kampot) started HEF in 2006/2008 and two ODs (KschaKandal and Takhmao) in Kandal since 2006/7 are supported by MoH, while the rest are supported by donor funds; the starting data of the HEF is between 2005 to 2011

**Table 2:** Characteristics of selected areas

	Ecological region	Province	No. of ODs	No. of RHs	No. of HCs	Total population	Characteristics
1.	Plain	Phnom Penh	4	5	17	1,327,615	Urban; high level external partner support
2.	Plain	Kandal	8	6	94	1,265,280	Rural; unsupported by partners
3.	Plain	Kampong Cham	10	11	136	1,679,992	Rural; supported by partners
4.	Tonle sap	Battambang	5	4	76	1,025,174	Rural; supported by partners
5.	Coastal	Kampot	4	4	50	585,850	Rural; supported by partners
6.	Plateau and mountainous	Stung Treng	1	1	11	111,671	Rural; supported by partners
Total for the country			77	80	292	13,395,682	

Source: document review for policies; NIS and MOP, 2009 for population data

For the DEA, DMUs are defined as operational districts. Each DMU is observed four times, providing panel data that can be used to assess productivity across the years and understand the impact of policy. Operational districts are treated as multi-product units producing a range of health services. The main services (outputs) were specified as numbers of inpatient-days, outpatients and deliveries. Inpatient days and outpatients provide a general measure of overall workload while deliveries are included because of the policy priority placed on increasing facility births. Data for outputs were drawn from national health statistics, which have been managed electronically since 2008.

Data on inputs were available on staffing numbers by type (doctors, secondary nurses, primary nurses, secondary midwives, primary midwives and other staff) and the non-staffing recurrent expenditure (Table 3). Staff numbers are collapsed into three categories: doctors, nurses & midwives and other staff. Inputs and outputs at facility level are aggregated to operational districts, which are the unit of analysis for the quantitative analysis. Expenditure data were missing for Kompot region and we therefore estimated efficiency for the full sample (108 data points) without expenditure and a restricted sample with expenditure (92 data points without Kompot).

**Table 3:** Average staffing in each operational district

	Battambang	Kampang Cham	Kampot	Kandal	Stung Treng
2008					
Doctors	6.8	6.0	8.3	11.4	10.0
Secondary nurse	62.2	37.2	42.8	21.6	59.0
Primary nurse	37.0	21.0	28.0	20.1	65.0
Secondary midwife	32.4	10.6	14.8	9.1	31.0
Primary midwife	19.2	12.1	15.8	11.8	42.0
Other medical staff	14.0	6.8	6.5	7.4	11.0
2011					
Doctors	5.8	6.6	9.5	12.4	9.0
Secondary nurse	62.0	37.8	41.3	22.9	68.0
Primary nurse	36.4	23.4	32.3	18.0	67.0
Secondary midwife	34.8	11.3	19.8	11.4	30.0
Primary midwife	23.0	19.8	22.3	15.8	50.0
Other medical staff	14.2	5.9	5.8	6.6	7.0

Other medical staff include pharmacists, pharmacist assistances and medical and dental assistants

The linear programming technique of Data Envelopment Analysis (DEA) is used to obtain output oriented estimates of production efficiency across operational districts. For constant returns to scale DEA solves the optimisation problem by maximising, for DMU (1), the e weighted (W_r_) sum of *N* outputs (O_r_):

$$\mathop \sum \limits_{r = 1}^{n} W_{r} O_{r1} ,\,\,\,\,\,\,{\text{where}}\,W_{r} \ge 0,$$

and subject to the constraint that the sum of the weighted Sum of M inputs is equal to one (to avoid an infinite number of solutions):

$$\mathop \sum \limits_{i = 1}^{m} V_{i} I_{i1} = 1,$$

and ensuring that all P DMUs have efficiency indices less than or equal to one:

$$\mathop \sum \limits_{r = 1}^{n} W_{r} O_{rj} - \mathop \sum \limits_{i = 1}^{m} V_{i} I_{ij} \le 0 j = 1, \ldots P.$$

There is no reason to assume the all operational districts will be operating at optimal scale and so both variable (VRS) as well as constant returns to scale (CRS) DEA efficiency scores are computed. While CRS assumes that an increase in inputs will increase outputs in the same proportion, VRS allows for a disproportionate change.

The data on operational districts is available for 4 years forming a balanced panel that permits consideration of the change in productivity over years. A Malmquist Productivity Index (MPI) is used for this purpose [25]. This provides a measure of the change in efficiency for each operational district from year to year. It is specified as the geometric mean of the index in each year. For DMU (1) this is:

$$MPI_{1, t + 1} = \left[ {\frac{{d_{1}^{t} \left( {I_{t + 1} ,O_{t + 1} } \right)}}{{d_{1}^{t} \left( {I_{t} ,O_{t} } \right)}} \times \frac{{d_{1}^{t + 1} \left( {I_{t + 1} ,O_{t + 1} } \right)}}{{d_{1}^{t + 1} \left( {I_{t} ,O_{t} } \right)}}} \right]^{0.5}$$

for all inputs (I) and outputs (O). A decomposition shows that the MPI is the product of changes associated with improvements in the technical efficiency of individual operational districts (getting closer to the industry frontier) and efficiency changes related to technical progress in the industry (technological efficiency resulting in a shift in the production frontier):

$$MPI_{1, t + 1} = \frac{{d_{1}^{t + 1} \left( {I_{t + 1} ,O_{t + 1} } \right)}}{{d_{1}^{t} \left( {I_{t} ,O_{t} } \right)}} \times \left[ {\frac{{d_{1}^{t} \left( {I_{t + 1} ,O_{t + 1} } \right)}}{{d_{1}^{t + 1} \left( {I_{t + 1} ,O_{t + 1} } \right)}} \times \frac{{d_{1}^{t} \left( {I_{t} ,O_{t} } \right)}}{{d_{1}^{t + 1} \left( {I_{t} ,O_{t} } \right)}}} \right]^{0.5}$$

An index (total or individual components) of more than one indicates positive growth from 1 year to the next an index less than one indicates negative growth.

DEA and MPI estimates were obtained using the Data Envelopment Analysis Programme (DEAP) developed by Coelli [26]. This accepts data formatted as text files structured with columns outputs and then input. Changes in efficiency can be explored further by attempting to understand the association between the score and possible determinants such as population density and poverty levels. Associations with the quality of the output (outcomes) can also be undertaken if data are available, which was not the case for this dataset. The results have, however, been triangulated with the qualitative data obtained from health mangers through key informant interviews (KII) and health workers consulted during the in-depth interviews (IDI), in order to contextualise and understand explanatory factors behind the quantitative results. Seventeen KIIs and 19 IDIs were conducted across five provinces between August and December 2013, using a semi-structure interview guide. Transcripts were analysed thematically [27]. Ethical approval was provided by the study by the Liverpool School of Tropical Medicine and the National Ethical Committee for Health Research of the Ministry of Health in Cambodia in 2012. Informed consent was provided by all participating health staff and managers.

The association between the DEA productivity index (with and without expenditure) and the presence of major health financing policies—health equity funds, vouchers and special operating areas—was also investigated. The index is bounded between zero and one (left and right censoring) and this truncation means that OLS estimators are inconsistent. Instead a random effects Tobit model, which provides consistent regression coefficients, is used to estimate the two specifications. The first regression explores the association between policies and productivity (E) as follows:

$$E_{it} = \alpha_{0} + \alpha_{1} t + \alpha_{2} P_{sit} + \alpha_{3} D_{i} + \alpha_{4} A_{i} + u_{st} + v_{i}$$

Where *t* is annual dummy variable, $P_{sit}$ are a series of dummy variables representing the main health financing policy combinations, D is the population density and A is regional dummy variable. The random effects model leads to a combination error term where $u_{st }$ is a district/time effect and $v_{i}$ an observation specific effect. A positive association suggests that areas with higher productivity are associated with the presence of health policies. It is, however, difficult to attribute causation since it is not possible to tell whether those areas with more productive services are more likely to be chosen to implement new financing policies. A second regression, attempts to look at whether the introduction of financing policies was associated with a change in productivity as follows:

$$E_{it} = \beta_{0} + \beta_{1} t + \beta_{2} P_{si}^{o} + \beta_{3} P_{it}^{n} + \beta_{4} D_{i} + \beta_{5} A_{i} + u_{st} + v_{i}$$

where $P_{si}^{o}$ is district specific variable for the main financing policies (HEF) and Vouchers and $P_{it}^{n}$ is a time/district specific dummy variable for the introduction of any major new financing policy. Significant association makes a causal link more likely although it still may have been possible for another variable to have improved both productivity and the introduction of health financing polices. The productivity DEA variable is constrained to take a value of between zero and one.

## Results and discussion

**Fig. 1:**
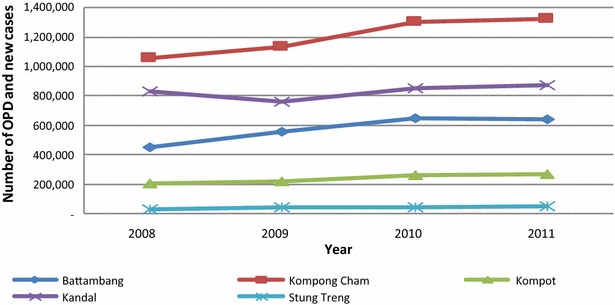
Trends in outpatient consultation and new cases by study province, 2008–2011 (Source: MoH’ s annual statistic report 2008–2011)

**Fig. 2:**
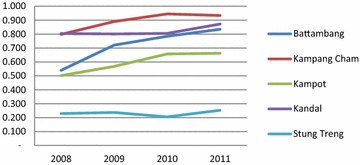
The results of efficiency scores without expenditures, 2008–2011

**Fig. 3:**
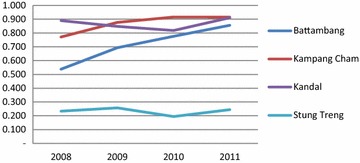
The results of efficiency scores with expenditures, 2008–2011

Utilisation of public health services in each province has increased over the 4 year period, as indicated by increasing trends in outpatients and number of new cases (aggregated in Fig. 1). The DEA results suggest great variation in the efficiency scores and trends of scores of public health services across the five provinces (Figs. 2, 3; detailed OD figures are provided in Additional file 1: Web Annex S1). Starting points were significantly different, but three of the five provinces have improved efficiency considerably over the period. Stung Treng remains a low performer throughout. These results are reinforced by the MPI estimates of productivity change (Table 4). These show low positive or negative productivity growth in Stung Treng while there is a strong and consistent improvement in productivity in other provinces, notably Battambang. The decompositions suggest that changes in productivity are evenly split between technical progress across the sector and improvements in individual efficiency resulting from economies of scale and better use of inputs.

**Table 4:** Malmquist index and decomposition (with and without expenditure as an input)

	Without expenditure				With expenditure			
	2008–2009	2009–2010	2010–2011	Mean	2008–2009	2009–2010	2010–2011	Mean
Marmquist productivity index								
Battambang	1.400	1.390	1.073	1.248	1.285	1.392	1.092	1.219
Kampang Cham	1.233	1.190	0.938	1.091	1.180	1.190	0.924	1.068
Kampot	1.242	1.193	0.916	1.053				
Kandal	1.046	1.126	0.998	1.042	1.019	1.075	1.028	1.025
Stung Treng	0.817	1.106	1.170	1.019	0.689	1.020	1.170	0.937
Total	1.175	1.233	0.974	1.092	1.128	1.186	1.004	1.080
Technical progress in sector								
Battambang	1.167	1.149	1.087	1.127	1.123	1.139	1.057	1.100
Kampang Cham	1.149	1.075	0.977	1.058	1.109	1.049	0.969	1.035
Kampot	0.978	1.046	0.930	0.975				
Kandal	1.137	1.042	0.937	1.033	1.079	1.015	0.959	1.014
Stung Treng	0.909	1.243	0.957	1.026	0.872	1.025	1.024	0.971
Total	1.121	1.095	0.985	1.059	1.092	1.055	0.987	1.039
Technical efficiency change								
Battambang	1.170	1.244	0.986	1.109	1.111	1.256	1.033	1.110
Kampang Cham	1.074	1.112	0.957	1.032	1.072	1.140	0.949	1.033
Kampot	1.289	1.183	0.984	1.082				
Kandal	0.922	1.083	1.063	1.009	0.949	1.060	1.071	1.011
Stung Treng	0.899	0.890	1.223	0.993	0.790	0.996	1.143	0.965
Total	1.045	1.137	0.989	1.031	1.027	1.132	1.015	1.039

It is perhaps not surprising that Kampong Cham, which is the only one of the provinces in this group to have been given SOA status, was one of the most efficient performers at the start and has increased its performance over the period (though less significantly if expenditures are taken into account, reflecting perhaps the higher payments which SOA areas receive). Five of its ten ODs have been given SOA status since 2009, which reflected its existing capacity. It also implemented the full range of demand- and supply-side stimuli (Table 1). It recorded the highest productivity growth throughput the period including between 2010 and 2011 when growth dipped for other provinces.

Kandal province, which has only implemented user fee formalisation on the demand side in an earlier phase and the midwifery incentive scheme in this phase across its ODs, and has had less external support as a province, nevertheless maintained a comparable efficiency to Kampang Cham, especially once expenditures are included (Fig. 2). Battambang started implementing delivery vouchers for three ODs in 2008 and scaled up health equity funds, staring in 2006 and reaching full implementation in all five ODs in 2011. It shows a robust increase in OD productivity, noted by a sharp raise of both efficiency scores with and without expenditures over the 4 year period.

The productivity of public health services in Kampot appears to level off and growth is negative between 2010 and 2011. The change in productivity is likely to come from the recent financial incentive schemes (vouchers, user fees, HEF/CBHI and NMIS) supported by government and donors funds to the ODs in Kampot Province (Table 1).

Among the five provinces, Stung Treng province shows the poorest performance, indicated by a stagnation of efficiency scores and low productivity growth, both with and without expenditures. This is likely to result from local conditions, as the area is mountainous and access to health services is constrained by poor roads, low population density and dispersed settlements of Cambodian ethnic minorities.

The regression analysis enables investigation of the association between performance and policy implementation. Each model was estimated with the CRS and VRS efficiency estimates, with and without expenditure as an input variable. Results reported here are for the entire sample and so exclude expenditure but similar results were found for the sub-sample that includes expenditure. The first model investigates the association between policy combinations and area covariates for both DEA indices with and without health spending (Table 5). Efficiency appears to increase over time (compared to the base year of 2008). In the CRS model, population density is associated with higher productivity, possibly reflecting the difficulty of reaching services for more remote populations. Areas with health equity funds in SOA and non-SOA areas are associated with higher productivity. The same effect is not found in areas only operating voucher schemes. The effect of the health equity funds are not found to be statistically significant in the VRS model, suggesting that lower efficiency in areas without equity funds is largely due to the facilities operating below optimum scale. The stimulus in use of service due to health equity funds helps facilities to achieve scale economies. The disappearance of a significant association with the densest populated areas is likely due to a similar mechanism (with facilities more able to achieve scale economies in highly populated areas).

**Table 5:** Tobit regression results for OD efficiency and policy/area characteristics (CRS and VRS, without expenditure)

	CRS		VRS		
	Coef.	z	Coef.	z	
Year					
2009	0.08	2.24*	0.11	2.41**	
2010	0.17	4.76	0.13	2.77***	
2011	0.15	4.11***	0.11	2.41**	
Policies					
Vouchers only	0.04	0.41	−0.05	−0.40	
HEF only	0.11	1.74**	−0.03	−0.40	
HEF + vouchers	0.16	2.24**	0.00	0.05	
HEF + vouchers with SOA	0.26	2.99***	0.16	1.52	
Population Density					
2	0.01	0.09	−0.02	−0.29	
3	−0.04	−0.23	−0.03	−0.18	
4	0.16	1.45**	0.09	0.74	
Province					
Kampong Cham	0.16	1.88***	0.14	1.66	
Kampot			−0.19	−1.97	
Kandal	0.11	1.29***	0.12	1.37	
Stung Treng	−0.27	−1.76	−0.25	−1.62	
Constant	0.46	5.45***	0.69	7.90	
Observations	92.00		92.00		

* *p* < 0.1, ** *p* < 0.05, *** *p* < 0.01

The second model (Table 6) attempts to disentangle the underlying association with financing schemes and any additional effect arising from the introduction of these schemes. Both CRS and VRS specifications suggest an increase in efficiency following the introduction of any or a combination of the three main policies; the efficiency score increases by between 0.11 and 0.12 the year any of the policies was introduced. It is notable that the policies have an impact in both CRS and VRS specifications. This suggests that the policies have an impact that goes beyond heavier utilisation of services resulting from the stimulus to demand.

**Table 6:** Tobit regressions: OD productivity and additional effects from new scheme introduction (CRS and VRS, without expenditure)

	CRS		VRS	
	Coef.	z	Coef.	z
Year				
2009	0.07	2.00**	0.09	2.02*
2010	0.17	4.56***	0.11	2.47***
2011	0.14	3.59***	0.09	1.85****
Policies				
Existing hef	0.06	0.87	−0.06	−0.92
Existing vouchers	0.09	1.17	0.08	1.02
New policy (hef,voucher, SOA)	0.114	2.41***	0.124	2.18**
Population density				
2	−0.02	−0.30	−0.04	−0.50
3	−0.08	−0.51	−0.05	−0.30
4	0.17	1.47*	0.10	0.89
Province				
Kampong Cham	0.18	2.17***	0.16	1.84*
Kampot	−0.17	−1.60*	−0.14	−1.35
Kandal	0.13	1.55	0.16	1.82*
Stung Treng	−0.19	−1.24	−0.18	−1.14
Constant				
Observations	92	–	92	–

* *p* < 0.1, ** *p* < 0.05, *** *p* < 0.01

### Explaining differences across ODs and provinces

It is unsurprising that population density emerges as a significant determinant of efficiency. Of the study provinces, Kandal and Kampong Cham had the highest population density in 2008, at 302 and 164 persons per square kilometre respectively [28] and the highest population to health worker (doctor, nurse and midwife) ratio of 1560 started with highest efficiency scores at around 0.8 in 2008. By contrast, Battambang province with 88 persons per square kilometre and 833 persons per health worker began at around 0.54 scores (Fig. 1). Stung Treng, with the lowest population density of 10 persons per square kilometre and 518 persons per health worker, had the lowest productivity among the five provinces.

Net immigration of population [29] seems to be associated with the rise of efficiency scores in the case of Kompong Cham between 2008 and 2011, Kompot for the period of 2008–2010, and Kandal province between 2010 and 2011. Population movement from other parts of the country is cited by the health managers of the hospitals and health centres as one factor in increasing demand.

For Stung Treng, at least two additional factors besides dispersed population and difficulty of access to the health facilities may have played a part in the region’s relatively low performance. First, there is less demand for health care among indigenous population who had limited knowledge of the importance of health care, and largely still used traditional midwives or healers, according to the health managers consulted, despite an increase in quality of health care and increased availability of services at the health facilities. Lack of trust in the public health service is another factor which was highlighted. Strung Treng is one of the least developed provinces in Cambodia, and is not favoured by the health workers. If they accept a posting there, according to our interviews, they do it just to complete the probationary period, and most of them, except the ones who are from this province, then ask for transfers to work elsewhere, or else they remain working in the provincial town of Stung Treng [27]. The young profile of staff in the province may cause a lack of confidence by households, and facilities such as ambulances for referral are reported in interviews as lacking.

“…It is difficult for us due to our age and experience in maternal health care. We don’t get much trust from the customers or patients. Customers always complained about our HC to let young midwives with less experience to treat them, especially when the problem happens…” (STIDI1)

In some areas, like Battambang, there is a thriving private sector which draws customers away from public facilities, which are perceived to offer lower quality.

On the supply side, budget disbursement and staffing problems can adversely affect facility functioning.

In Kompot province, for example, our interviews found that budget disbursement to facilities was often delayed and reduced. Another more general issue was mal-distribution of staff and the fact that actual staffing numbers were lower than those officially recorded. Mal-distribution of health workers reduces efficiency. The transfer of qualified and experienced health workers from the least to the most developed areas is a widespread problem, which leaves the public health facilities in rural areas with poorly qualified staff. Thus, the regular presence of health workers in the public facilities may not lead to an increase in production of health service delivery or utilisation of the public health services, especially in the rural areas. Despite receiving financial incentives in recent years, including from user fees and midwifery incentives, as well as an automatic annual salary increase of 20 % since 2010/11, deploying and retaining health workers to work in the rural area remains a critical challenge for OD managers. Field visits also suggest a lack of equipment, especially in health centres and rural areas.

### Explaining the policy effects

The recent health financing reforms have aimed to stimulate supply and demand, and our results suggest that they have been effective, singly and in combination. This is compatible with results of evaluations of specific policies. For example, Chhun et al. found that that HEFs and vouchers increased health care access of the poor at both public and private facilities [30]. On the supply side, interviews with health workers and managers [27] also highlight that a large proportion of income collected from the financial incentive schemes was used for increasing the income and motivation of health workers, as well as being used by the facility managers to make the service at the public facilities available for 24 h a day to the population.

In Kampong Cham, three factors may explain its relatively higher efficiency scores. First, it has implemented the full range of demand-side and supply-side interventions. Secondly, as an early adopter, it benefited from management capacity development in the early stages of the pilot schemes—experiences which have been shared among the facility managers through quarterly provincial health department (PHD) meetings to improve their leadership and management skills, according to key informants. Finally, NGO support since 2000 in improving health infrastructure and supply of medical equipment at facility level is thought to be in part responsible for increased capacity for service delivery.

### Health worker explanatory factors

One of the factors behind low efficiency is the limited working hours of many public sector workers. From in-depth interviews with health workers, these are estimated as varying between 3 h for well-experienced specialist doctors to 8 h for newly graduated midwife; and on average, the secondary level cadres are likely to spend at least 6 h working at their public posts.

The revenue collected from the demand side financial schemes is reportedly put into pooled income of the facility; and 60 % of this income is then distributed among the active staff. The health workers earn a supplementary income of around 100–150 USD per month, according to their level of education attainment and the types of tasks they accomplish, in addition to their basic salary of about 65USD and an annual increase 20 % of basic salary from the government for the past 5 years [31]. However, most mangers and health workers argued that income from public services remains lower than the expenditure that they need to support their families. Therefore, the health managers felt that:

“… I cannot force my staff to work full time, although they receive a top up average of 100-150USD per month from recent incentive based payment schemes in addition to their salary. But it cannot off set what they need to spend to support their families. Therefore, I often open one eye and close another eye when my staff come late at work or leave a bit early for making living to support their families. My task then is to ensure the quality of worker when the staff present at their post…” (KCII1)

In the health sector, the health workers often need to do dual practice to make extra income to support their family. For example, renting a house costs around 100USD per month, depending on the type of house; this is already higher than the average salary of a public health worker. The opportunity for informal practice or taking part time jobs is identified by most health managers as an important, though illegal, strategy to motivate the health workers to remain in their posts. One of the managers estimated that dual practice has reportedly contributed about 40–50 % of staff revenues and most managers agreed that:

“… if we completely stop the informal practice, I am sure they will all have gone” (KCII2).

Low salaries reduce public sector input costs, but may encourage dual practice which tends to reduce public sector (but not necessarily overall) outputs. Unfortunately, we lack trend data for overall public pay and dual practice over the period, to be able to disentangle effects on efficiency measures. However, it is important to bear in mind that most of health workers set up their private businesses (clinics) or take part-time employment at private clinics or provided private service to their clients for extra income generation to support their family. A recent study confirmed that over 50 % of the public health workers interviewed also worked in the private sector in 2012 [32]. Overall human resource productivity will therefore be higher than reported here, but with additional costs to consumers.

In the civil code of professional conduct, public health workers are required to work for 8 h a day and are not allowed to have dual employment. In practice, the managers cannot enforce punctuality and stop private practice after-office hours, and they often report a sympathetic management practice to retain key health personnel, especially the ones with technical competency. This management practice has resulted in positive and negative effects on the quality of public health service delivery [27]. On the positive side, it makes health care service available for 24 h by rotating stand-by staff, with urgent calls to specific health professionals if it is necessary. The negative side of this sympathetic management practice is the diversion of clients to private clinics or individual home care, which affects the efficiency scores of most public health care services captured by this study.

### Reflections on data reliability

It is important to reflect on how accurate the staffing numbers are and whether staff are actually working their official hours, both of which affect the efficiency estimates. Triangulating with qualitative information suggests that fewer health workers actually remain working in post than officially recorded by the MoH. One of the in-depth interview participants working in a remote health centre confirmed in Stung Treng that:

…There are four staffs at this HC, but only one or two are active. I am working here, there is no replacement. For my unit, there are only 3 staff and one is active while others come and go… (STIDI2).

The OD and health centre mangers report that they have often received the list of the names of the newly recruited staffs from the Ministry of Health or Provincial Health Department in response to the requests they have put forward in the human resource plan. While the list of personnel is officially updated, some new health workers such as doctors or secondary nurses or midwifes had never shown up at their assigned posts, or if they do, they do not go regularly to their assigned posts. With interventions from the high-ranking officials and other family connections, they then ask to be transferred to work elsewhere after completing 1 year of their probation period. In these cases, the name of those staff can remain in the official records for 6–12 months, before they are corrected.

Some experienced health workers have taken leave without pay from the public sector to work for NGO health-related programmes or private providers, and their names may reportedly remain in the official records. Such shortfalls can be addressed in areas with internal contracting and SOA status, but in others it is harder to fill these gaps, according to health manager key informants at all levels of the health system.

It should be noted too that health workers at the OD level also take on other activities which were not reflected in our efficiency analysis (such as village outreach visits), due to lack of data on these activities.

## Conclusion

Our analysis suggests increased efficiency in most of the selected ODs over the period, but with substantial variations across the provinces. Some important limitations are however noted, including data shortages, which reduced the number of sites and the type of analysis which could be conducted. Quality of care indicators are absent, some information (such as staffing numbers) may not be fully accurate, and important information is not included, such as working hours and public pay. Also, while it is innovative to aggregate inputs and outputs for all facilities in a health district, the results must be interpreted with care: by aggregating, efficient facilities will be fused with less efficient ones. Thus, the fact that a health district is inefficient does not mean that it has no efficient facilities. The findings do however open up a series of questions, which qualitative information complements. For example, the difficult working and access conditions in Stung Treng suggest that a different set of interventions might be needed there to boost outputs, compared to other areas. Addressing efficiency requires an understanding of area factors, organisational level factors and individual supply-and demand-side factors, as well as interaction between public, private and informal markets. Some of these are more amenable to policy levers than others.

In Cambodia, this is the first study of OD productivity, and indeed it adds to a very limited body of evidence on health district efficiency (most studies which have been done in low and middle income settings have analysed the hospital as the unit of production), and on the impact of health sector reforms on productivity. A number of policy implications can be drawn from this study. For effective resource planning and monitoring purposes, the administrative records should be improved. It would also be interesting and useful to replicate the analysis using country-wide data sets. The results of this country-wide analysis could be used for resource planning and targeting of new initiatives. The finding of an association between recent policy interventions and improved productivity of public health services will also be of interest to other countries planning similar health sector reforms.

## Additional file

10.1186/s12962-016-0051-6 Efficiency scores by operational district.
