# Supplementary material for: Exploring the influence of context and policy on health district productivity in Cambodia
Source: Cost Eff Resour Alloc. 2016 Jan 22;14:1. doi: 10.1186/s12962-016-0051-6 (PMC4724134; doi:10.1186/s12962-016-0051-6)
Supplement: Supplementary file 1 — 10.1186/s12962-016-0051-6 Efficiency scores by operational district. [file 12962_2016_51_MOESM1_ESM.docx]

**Web annex 1: Efficiency scores by operational district**

|  |  | Without expenditures | | | | With expenditures | | | |
| --- | --- | --- | --- | --- | --- | --- | --- | --- | --- |
| Province | OD | 2008 | 2009 | 2010 | 2011 | 2008 | 2009 | 2010 | 2011 |
| Battambang | Battambang | 0.9242 | 0.8674 | 1 | 1 | 0.7774 | 0.8225 | 1 | 1 |
| Battambang | Mong Russey | 0.4485 | 0.5916 | 0.7776 | 0.7831 | 0.5385 | 0.5938 | 0.8285 | 0.8612 |
| Battambang | Sampov Luon | 0.4765 | 1 | 0.8503 | 1 | 0.4039 | 1 | 0.7426 | 1 |
| Battambang | Sangkae | 0.505 | 0.7132 | 0.7237 | 0.7211 | 0.5694 | 0.621 | 0.7314 | 0.7116 |
| Battambang | Thmor Koul | 0.3415 | 0.4342 | 0.5695 | 0.6733 | 0.4013 | 0.4317 | 0.5779 | 0.7085 |
| Kampang Cham | Chamkar leu-Stueng Trang | 0.7937 | 1 | 0.9452 | 1 | 0.6873 | 1 | 0.9425 | 1 |
| Kampang Cham | Choeung Prey-Batheay | 0.7949 | 1 | 1 | 1 | 0.7698 | 1 | 1 | 1 |
| Kampang Cham | Kampong Cham-Kampong Siem | 1 | 1 | 1 | 1 | 1 | 1 | 0.8879 | 1 |
| Kampang Cham | Kroch Chhmar-Stueng Trang | 0.5884 | 0.3537 | 0.4911 | 0.4789 | 0.5577 | 0.334 | 0.6275 | 0.563 |
| Kampang Cham | Memot | 0.831 | 1 | 1 | 1 | 0.8396 | 1 | 1 | 1 |
| Kampang Cham | O Reang Ov-Kaoh Soutin | 0.531 | 0.6636 | 0.8936 | 1 | 0.5197 | 0.6221 | 0.8471 | 0.9716 |
| Kampang Cham | Ponhea Krek-Dambae | 0.9432 | 1 | 1 | 1 | 0.9234 | 1 | 1 | 1 |
| Kampang Cham | Prey Chhor-Kang Meas | 0.929 | 1 | 1 | 1 | 0.9886 | 1 | 1 | 1 |
| Kampang Cham | Srei Santhor-Kang Meas | 0.8316 | 0.9098 | 0.9264 | 0.6224 | 0.7556 | 0.883 | 0.9036 | 0.6189 |
| Kampang Cham | Tbong Khmum-Kroch Chhmar | 0.5919 | 0.8299 | 1 | 1 | 0.6696 | 0.9261 | 0.9468 | 1 |
| Kampot | Angkor Chey | 0.4011 | 0.7206 | 0.7134 | 0.7258 |  |  |  |  |
| Kampot | Chhouk | 0.3868 | 0.5445 | 0.707 | 0.7753 |  |  |  |  |
| Kampot | Kampong Trach | 0.6547 | 0.6426 | 0.7681 | 0.6829 |  |  |  |  |
| Kampot | Kampot | 0.5638 | 0.3653 | 0.4399 | 0.4647 |  |  |  |  |
| Kandal | Ang Snuol | 0.6941 | 0.6491 | 0.6859 | 0.9268 | 0.9865 | 0.8759 | 0.7229 | 1 |
| Kandal | Kean Svay | 0.6658 | 0.8072 | 0.9343 | 1 | 0.7244 | 0.8895 | 0.909 | 1 |
| Kandal | Koh Thom | 0.8714 | 1 | 1 | 1 | 0.9462 | 1 | 1 | 1 |
| Kandal | Ksach Kandal | 0.9797 | 1 | 0.9613 | 1 | 0.9149 | 0.9523 | 0.9083 | 0.9751 |
| Kandal | Muk Kam Poul | 0.8037 | 0.6422 | 0.841 | 0.8663 | 0.8778 | 0.6273 | 0.7581 | 0.8487 |
| Kandal | Ponhea Leu | 0.7567 | 0.5684 | 0.5117 | 0.5416 | 0.9157 | 0.6131 | 0.585 | 0.6837 |
| Kandal | Saang | 0.6604 | 0.7425 | 0.7688 | 0.773 | 0.7504 | 0.8249 | 0.8133 | 0.819 |
| Kandal | Takhmau | 1 | 0.9984 | 0.7416 | 0.8711 | 1 | 1 | 0.8465 | 0.9642 |
| Kampang Cham | OD Center | 1 | 0.7332 | 0.976 | 0.9752 |  |  |  |  |
| Kampang Cham | OD North | 0.7088 | 1 | 1 | 1 |  |  |  |  |
| Kampang Cham | OD South | 0.7977 | 1 | 1 | 1 |  |  |  |  |
| Kampang Cham | OD West | 0.8347 | 0.9768 | 1 | 1 |  |  |  |  |
| Stung Treng | Stung Treng | 0.2292 | 0.2366 | 0.2053 | 0.2524 | 0.2334 | 0.2577 | 0.1953 | 0.2447 |
